# Association between the FT3/FT4 ratio and thyroid nodules in euthyroid patients with type 2 diabetes: a cross-sectional study

**DOI:** 10.3389/fmed.2026.1908557

**Published:** 2026-10-20

**Authors:** Bo Huang, Xin Li, Dadi Xie, Xin-Xin Zhang, Shi-Wei Li, Yu-Xin Tian, Chao-Yi Li, Jing-Qiu Cui

**Affiliations:** 1 Department of Endocrinology and Metabolism, Tianjin Medical University General Hospital, Tianjin, China; 2 Department of Endocrinology and Metabolism, Tengzhou Central People’s Hospital, Shandong, China; 3 Department of Nephrology, Tianjin Medical University General Hospita, Tianjin, China

**Keywords:** euthyroidism, FT3, FT3/FT4 ratio, thyroid nodule, type 2 diabetes

## Abstract

The association between the free triiodothyronine-to-free thyroxine (FT3/FT4) ratio and thyroid nodules (TNs) remains unclear. This cross-sectional study examined this association in euthyroid patients with type 2 diabetes mellitus (T2DM). Between 2021 and 2023, we enrolled 934 patients with T2DM at Tianjin Medical University General Hospital. All participants underwent anthropometric assessment, physical examination, laboratory testing, and thyroid ultrasonography. Logistic regression models were used to evaluate the association between the FT3/FT4 ratio and TNs. Restricted cubic spline analysis was performed to explore potential nonlinearity. Overall, 730 participants (78.2%) had TNs. The prevalence was higher in women than in men (84.2% vs. 73.9%). Participants with TNs had lower 24-h urinary uric acid excretion, estimated glomerular filtration rate, glycated hemoglobin, and FT3/FT4 ratio than those without TNs. The between-group difference in the FT3/FT4 ratio was statistically significant (*P* = 0.027). In the unadjusted analysis, a higher FT3/FT4 ratio was associated with lower odds of TNs. However, the association was attenuated after adjustment for age and sex and remained nonsignificant after further adjustment for clinically relevant covariates. Restricted cubic spline analysis indicated a significant nonlinear association between the FT3/FT4 ratio and TNs (*P* for overall association = 0.036; *P* for nonlinearity = 0.022), with higher estimated odds of TNs observed at the lower end of the FT3/FT4 ratio distribution. However, the 95% confidence intervals included 1 across most of the distribution. In euthyroid patients with T2DM, the FT3/FT4 ratio was not independently associated with TNs under a conventional linear assumption. However, the spline analysis suggested a possible nonlinear relationship that warrants confirmation in prospective studies.

## Introduction

Thyroid nodules (TNs) are common in the general population and are often detected incidentally on ultrasonography or during routine physical examinations, as most are asymptomatic (1). Although most TNs are benign, some may cause compressive symptoms or warrant further evaluation to exclude malignancy. A recent population-based study reported an overall prevalence of 36.9% in China, with substantial regional variation (2). Moreover, Zhang et al. reported a higher prevalence of TNs among individuals with type 2 diabetes mellitus (T2DM) than among those without diabetes (3). Given the high prevalence of TNs and the growing burden of T2DM, identifying factors associated with TNs in patients with T2DM may have clinical relevance.

The development of TNs is influenced by multiple factors, including genetic susceptibility, iodine status, thyroid hormone abnormalities, insulin resistance, and chronic inflammation (4). T2DM is characterized by insulin resistance and chronic low-grade inflammation, both of which may contribute to thyroid cell proliferation and nodule formation. Obesity and other metabolic abnormalities commonly associated with T2DM may also affect thyroid morphology and function. Collectively, these metabolic disturbances may partly account for the higher prevalence of TNs in individuals with T2DM.

Thyroid hormone homeostasis is regulated by the hypothalamic–pituitary–thyroid axis and the peripheral conversion of thyroxine (T4) to triiodothyronine (T3) (5). Several indices have been proposed to assess central or peripheral sensitivity to thyroid hormones. Among these, the sum activity of peripheral deiodinases (SPINA-GD) and the free triiodothyronine-to-free thyroxine (FT3/FT4) ratio are used as indirect measures of peripheral T4-to-T3 conversion (6–8). The FT3/FT4 ratio can be readily calculated from routinely measured thyroid function parameters and may reflect alterations in peripheral thyroid hormone metabolism. Previous studies have reported associations between thyroid hormone sensitivity indices and diabetes, metabolic syndrome, and obesity, even among euthyroid individuals (9–11).

However, the clinical relevance of altered peripheral thyroid hormone metabolism in thyroid nodular disease remains unclear. Li et al. reported that euthyroid men with TNs had a lower FT3/FT4 ratio (12), whereas, Sun et al. reported that a lower FT3/FT4 ratio was associated with papillary thyroid carcinoma (13). Evidence regarding the association between the FT3/FT4 ratio and TNs in euthyroid patients with T2DM remains limited. This study aimed to evaluate this association among hospitalized patients with T2DM.

## Methods and materials

### Subjects

In total, 934 T2DM patients who were admitted to the Tianjin Medical University General Hospital between 2021 and 2023 were included in the study (Figure 1). Every participant had a general medical examination that included laboratory testing, thyroid ultrasound examination, and anthropometric evaluations. The exclusion criteria were as follows: 1) patients younger than 18 years old or older than 80 years old, repeated hospitalization and incomplete information; 2) type 1 diabetes mellitus or other specific types of diabetes; 3) acute diabetic complications, inability to eat normally, or a history of severe hypoglycemia; 4) tumor diseases, and blood system diseases; 5) patients were excluded if they had a history of thyroid disease, including thyroid cancer, hypothyroidism, subclinical hypothyroidism, or hyperthyroidism; a history of thyroid surgery; positive thyroid-related antibodies; or use of thyroid-related medications or other medications known to affect thyroid function. Hyperuricemia was diagnosed in both men and females with SUA values 7.0 mg/dL (420 μmol/L) (14). Type 2 diabetes mellitus was diagnosed according to the 1999 World Health Organization diagnostic criteria (15). Hypertension was defined as a self-reported history of hypertension, systolic blood pressure (SBP) ≥ 140 mmHg, or diastolic blood pressure (DBP) ≥ 90 mmHg (16). Obesity was defined as a body mass index (BMI) ≥ 28 kg/m^2^ (17).

**Figure 1:**
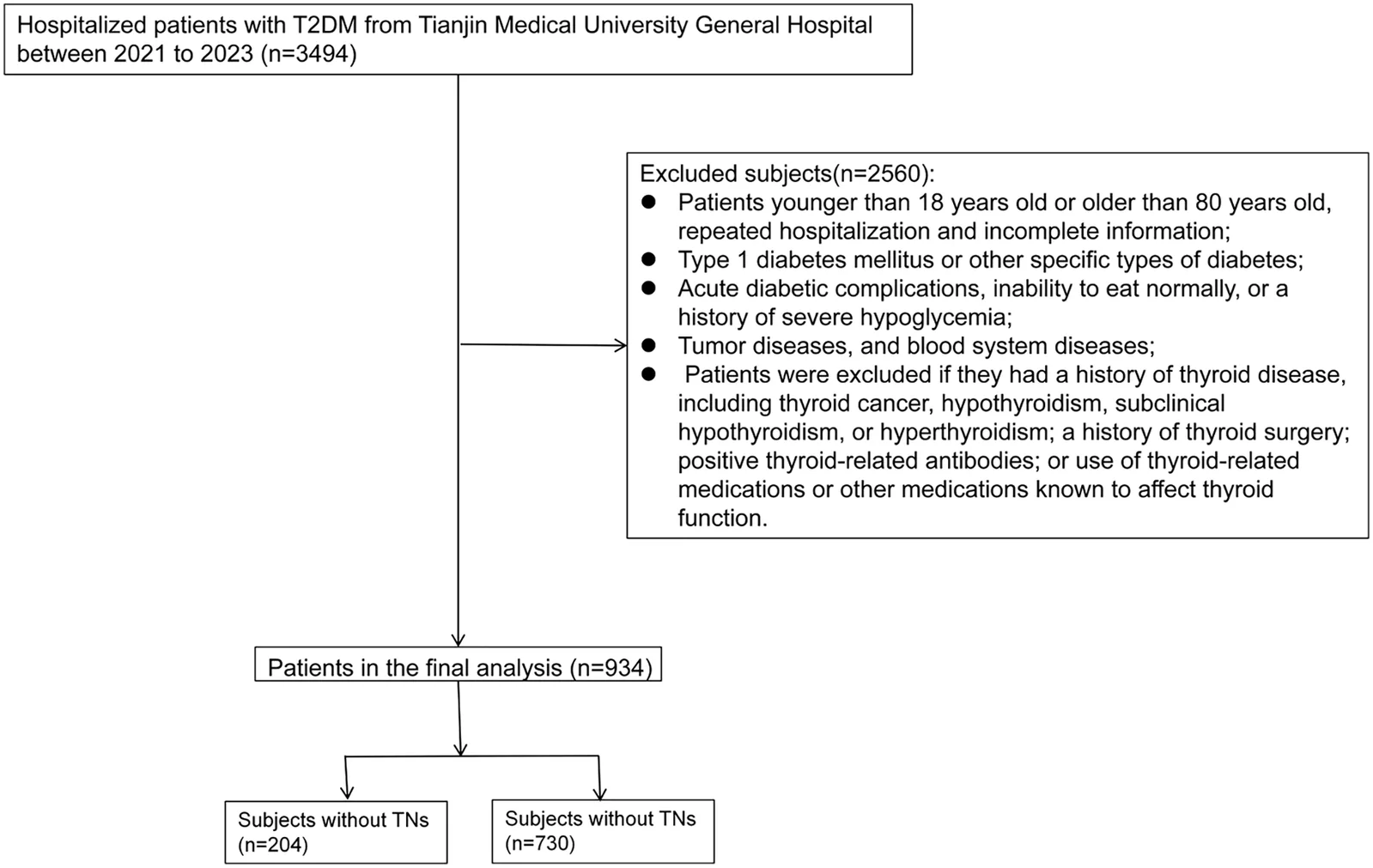
Flowchart of the cross-sectional study.

### Data collection

Demographic and clinical data were collected for all participants, including age, sex, year of birth, smoking and alcohol consumption status, duration of diabetes, family history of diabetes, and previous diagnoses of hyperuricemia (HUA), hypertension, diabetic retinopathy (DR), diabetic kidney disease (DKD), diabetic sensorimotor polyneuropathy (DSPN), carotid artery stenosis (CAS), and lower-extremity atherosclerotic disease (LEAD). Trained hospital staff measured height, weight, systolic blood pressure (SBP), and diastolic blood pressure (DBP).

Information on insulin and metformin use was collected and included in the multivariable analysis. Free triiodothyronine (FT3), free thyroxine (FT4), thyroid-stimulating hormone (TSH), serum creatinine (Cr), serum uric acid (SUA), estimated glomerular filtration rate (eGFR), fasting plasma glucose (FPG), total cholesterol (TC), triglycerides (TG), high-density lipoprotein cholesterol (HDL-C), low-density lipoprotein cholesterol (LDL-C), alanine aminotransferase (ALT), aspartate aminotransferase (AST), *γ*-glutamyl transferase (GGT), glycated hemoglobin A1c (HbA1c), and 24-h urinary uric acid excretion (UUAE) were measured using an automated analyzer. The laboratory reference ranges were 2.43–6.01 pmol/L for FT3, 9.01–19.05 pmol/L for FT4, and 0.35–4.96 μIU/mL for TSH. Euthyroidism was defined as FT3, FT4, and TSH concentrations within these reference ranges. The FT3/FT4 ratio was calculated by dividing FT3 (pmol/L) by FT4 (pmol/L) (18). Greater peripheral sensitivity to thyroid hormones is indicated by greater FT3/FT4 levels (19). Participants were categorized into quartiles according to the distribution of the FT3/FT4 ratio in the analytic cohort: Q1, ≤0.2691; Q2, >0.2691 to ≤0.2963; Q3, >0.2963 to ≤0.3249; and Q4, >0.3249.

Thyroid ultrasonography was performed by experienced thyroid specialists using a 10-MHz linear-array transducer. Nodule size, number, location, echogenicity, margins, cystic components, and Thyroid Imaging Reporting and Data System (TI-RADS) category were obtained from the original ultrasound reports. Participants were classified into groups with and without TNs based on the ultrasonographic findings. Because cytological or histopathological confirmation was not routinely available, TNs were defined by ultrasonography rather than pathological examination. Body mass index (BMI) was calculated as weight in kilograms divided by height in meters squared (kg/m^2^). The estimated glomerular filtration rate (eGFR) was calculated using the Chronic Kidney Disease Epidemiology Collaboration (CKD-EPI) equation (20).

### Statistical analysis

Data were analyzed using IBM SPSS Statistics, version 27. Normality was assessed using the Kolmogorov–Smirnov test. Categorical variables are presented as frequencies and percentages, whereas continuous variables are expressed as the mean ± standard deviation or median (interquartile range), as appropriate. Normally distributed variables were compared using independent-samples t-tests or one-way analysis of variance, and non-normally distributed variables using Mann–Whitney *U* or Kruskal–Wallis tests. Categorical variables were compared using the chi-square test.

Spearman correlation analysis was used to examine associations between the FT3/FT4 ratio and other variables. Its association with TNs was evaluated using binary logistic regression. Covariates were selected *a priori* based on clinical and biological relevance. Model 1 was unadjusted; Model 2 included age and sex; and Model 3 additionally included BMI, diabetes duration, smoking status, HbA1c, insulin use, metformin use and TSH. Results are reported as odds ratios with 95% confidence intervals.

## Results

### Baseline characteristics

Table 1 summarizes the clinical and biochemical characteristics of the study participants. Participants with TNs were older than those without TNs (*P* < 0.001) and had lower FT3/FT4 ratios, eGFR, FPG, TC, TG, LDL-C, ALT, AST, GGT, HbA1c, and UUAE levels (all *P* < 0.05). Hypertension and CAS were more prevalent among participants with TNs, whereas LEAD was less prevalent (all *P* < 0.001). Insulin and metformin use was also less common among participants with TNs (both *P* < 0.001).

**Table 1:** Characteristics of patients categorized by the presence of tns.

Parameters	TNs (-) (*n* = 204)	TNs (+) (*n* = 730)	*P*
male (%)	143 (70.10%)	405 (55.48%)	*p* < 0.001
Age (yr)	50.5 (40.6, 60.4)	62 (54, 70)	*p* < 0.001
FT3 (pmol/L)	3.83 (3.60, 4.06)	3.83 (3.56, 4.03)	0.272
FT4 (pmol/L)	12.74 (11.74, 13.74)	12.79 (11.79, 13.79)	0.275
TSH (μIU/mL)	1.52 (1.02, 2.02)	1.47 (0.97, 1.97)	0.200
FT3/FT4	0.303 ± 0.040	0.296 ± 0.041	0.025
Cr (μmol/L)	62 (50, 74)	62 (50.5, 73.5)	0.961
SUA (μmol/L)	344.5 (279.5, 409.5)	326 (257, 395)	0.076
eGFR (mL/min/1.73m2)	110.73 (96.03, 125.43)	104.66 (92.05, 117.27)	*p* < 0.001
FBG (mmol/L)	7.79 (5.79, 9.79)	7.2 (5.2, 9.2)	0.007
TC (mmol/L)	4.75 (3.75, 5.75)	4.54 (3.54, 5.54)	0.042
TG (mmol/L)	2.24 (1.24, 3.24)	1.81 (0.81, 2.81)	0.007
HDL (mmol/L)	0.99 (0.83, 1.14)	1.01 (0.85, 1.19)	0.133
LDL (mmol/L)	3.06 (2.56, 3.56)	2.76 (2.26, 3.26)	0.002
ALT (U/L)	24.5 (11, 38)	19 (11.5, 26.5)	*p* < 0.001
AST (U/L)	19 (12, 26)	18 (14, 22)	0.003
GGT (U/L)	29 (14, 44)	24 (15, 33)	*p* < 0.001
HbA1c (%)	8.9 (6.9, 10.9)	8.3 (6.8, 9.8)	0.007
UUAE (mg/24 h)	629.78 (459.28, 800.28)	586.24 (439.74, 732.74)	*p* < 0.001
SBP (mmHg)	130.5 (119, 142)	133 (122, 144)	0.097
DBP (mmHg)	80 (72, 88)	80 (73, 87)	0.131
BMI (kg/m2)	25.88 (23.27, 28.49)	25.64 (23.26, 28.01)	0.222
Obesity *n* (%)	60 (29.4%)	191 (26.2%)	0.355
Smoking *n* (%)	87 (42.6%)	277 (37.9%)	0.223
Drinking *n* (%)	76 (37.3%)	245 (33.6%)	0.326
DM family history *n* (%)	84 (41.2%)	258 (35.3%)	0.126
HUA *n* (%)	50 (24.5%)	168 (23%)	0.655
Hypertension *n* (%)	96 (47. 1%)	437 (59.9%)	0.001
DR *n* (%)	44 (21.6%)	190 (26%)	0.194
DKD *n* (%)	49 (24%)	217 (29.7%)	0.110
DSPN *n* (%)	51 (25%)	234 (32. 1%)	0.053
CAS *n* (%)	109 (53.4%)	496 (67.9%)	*p* < 0.001
LEAD *n* (%)	111 (54.4%)	216 (29.6%)	*p* < 0.001
Insulin *n* (%)	79 (38.7%)	140 (19.2%)	*p* < 0.001
Metformin *n* (%)	73 (35.8%)	140 (19.2%)	*p* < 0.001

FT3, free Triiodothyronine; FT4, free thyroxine; TSH, thyroid-stimulating hormone; Cr, creatinine; SUA, serum uric acid; eGFR, estimated glomerular filtration rate; FPG, fasting plasma levels of glucose; TC, total cholesterol; TG, triglyceride; HDL, high-density lipoprotein; LDL, low-density lipoprotein; ALT, alanine aminotransferase; AST, aspartate aminotransferase; GGT, glutamyl transpeptidase; HbA1c, glycated hemoglobin A1c; UUAE, 24 h urinary excretion of uric acid; SBP, systolic blood pressure; DBP, diastolic blood pressure; BMI, body mass index; DM, diabetes mellitus; HUA, hyperuricemia; DR, diabetic retinopathy; DKD, diabetic kidney disease; DSPN, diabetic sensorimotor polyneuropathy; CAS, carotid artery stenosis; LEA, lower extremity atherosclerosis.

Compared with Q1group, *p < 0.05; compared with Q2 group, #*p* < 0.05; compared with Q3 group, &*p* < 0.05.

Data are presented as number (percentage) for categorical variables and as median (with 25th and 75th percentiles) for continuous variables.

The general characteristics of the participants according to quartiles of the FT3/FT4 ratio are summarized in Table 2. Participants in Q3 and Q4 were younger and had higher BMI values than those in Q1 (all *P* < 0.05). As the FT3/FT4 quartile increased, FT3 levels increased, whereas FT4 levels decreased (both *P* < 0.05). Compared with Q1, participants in Q2–Q4 had higher eGFR and UUAE levels, while those in Q3 had lower serum creatinine levels (all *P* < 0.05). Compared with participants in Q1, those in Q4 had lower HbA1c levels and higher ALT and GGT levels (all *P* < 0.05). The prevalence of diabetic retinopathy and diabetic kidney disease also differed significantly across the quartiles (*P* = 0.044 and *P* = 0.041, respectively). In contrast, no significant differences were observed in TSH, SUA, TC, or LDL-C across the four quartiles (all *P* > 0.05).

**Table 2:** Characteristics of patients categorized by quartiles of FT3/FT4 ratio.

Parameters	Q1	Q2	Q3	Q4	*p*-value
Gender *n* (%)	120 (51.9%)	138 (59%)	144 (60.8%)	146 (62.9%)	0.091
Age (yr)	63 (56, 70)	61 (52, 70)	58 (48, 68)*	59 (49, 69)*	*p* < 0.05
FT3 (pmol/L)	3.5 (3, 4)	3.73 (3.73, 3.73)*	3.93 (3.93, 3.93)^*^^,^^#^	4.04 (4.04, 4.04)^*^^,^^#^^,^^&^	*p* < 0.05
FT4 (pmol/L)	14. 16 (13.16, 15. 16)	13.2 (12.7, 13.7)*	12.66 (12.16, 13. 16)^*^^,^^#^	11.57 (11.07, 12.07)^*^^,^^#^^,^^&^	*p* < 0.05
TSH (μIU/mL)	1.46 (0.96, 1.96)	1.43 (0.93, 1.93)	1.48 (0.98, 1.98)	1.57 (1.07, 2.07)	0.292
Cr (μmol/L)	66 (54.5, 77.5)	61 (49.5, 72.5)	61 (50.5, 71.5)*	62 (51, 73)	0.011
SUA (μmol/L)	331 (255, 407)	329.5 (257, 402)	329 (264.5, 393.5)	331 (265.5, 396.5)	0.780
eGFR (mL/min/1.73m2)	101.73 (86.72, 116.75)	106.21 (93.85, 118.57)*	108. 1 (95.52, 120.68)*	107.03 (94.86, 119.21)*	*p* < 0.05
FBG (mmol/L)	7.4 (5.4, 9.4)	7.65 (5.65, 9.65)	7.5 (5.5, 9.5)	6.95 (5.45, 8.45)^#^	0.027
TC (mmol/L)	4.68 (3.68, 5.68)	4.56 (3.56, 5.56)	4.61 (3.61, 5.61)	4.51 (3.51, 5.51)	0.246
TG (mmol/L)	1.79 (0.79, 2.79)	1.79 (0.79, 2.79)	1.94 (0.94, 2.94)	1.94 (0.94, 2.94)	0.108
HDL (mmol/L)	1.02 (1.02, 1.02)	1.02 (1.02, 1.02)	1.01 (1.01, 1.01)	0.98 (0.98, 0.98)	0.517
LDL (mmol/L)	2.79 (2.29, 3.29)	2.84 (2.34, 3.34)	2.95 (2.45, 3.45)	2.75 (2.25, 3.25)	0.208
ALT (U/L)	18 (11, 25)	20 (12, 28)	21 (10, 32)	22 (13.5, 30.5)*	0.017
AST (U/L)	18 (14, 22)	18 (13.5, 22.5)	17 (12, 22)	18 (14, 22)	0.830
GGT (U/L)	22 (13.5, 30.5)	25 (15, 35)	25 (12.5, 37.5)	27 (16.5, 37.5)*	0.005
HbA1c (%)	8.9 (6.9, 10.9)	8.9 (7.4, 10.4)	8.6 (7.1, 10. 1)	7.55 (6.55, 8.55)^*^^,^^#^^,^^&^	*p* < 0.05
UUAE (mg/24 h)	543.84 (398.34, 689.34)	593.64 (448.64, 738.64)*	628.54 (467.54, 789.54)*	607.2 (467.7, 746.7)*	*p* < 0.05
SBP (mmHg)	132 (120.5, 143.5)	133.5 (123.5, 143.5)	133 (122.5, 143.5)	130.5 (117.5, 143.5)	0.479
DBP (mmHg)	78 (71, 85)	80 (72.5, 87.5)	80 (73, 87)*	80.5 (72.5, 88.5)	0.030
BMI (kg/m2)	24.96 (22.43, 27.5)	25.63 (23.17, 28.08)	25.95 (23.18, 28.73)*	26. 17 (24.17, 28. 18)*	0.002
Obesity *n* (%)	47 (20.3%)	65 (27.8%)	72 (30.4%)	67 (28.9%)	0.070
Duration *n* (%)	10 (1, 19)	10 (2.38, 17.63)	9 (2, 16)	7 (0.5, 13.5)	0.064
Smoking *n* (%)	79 (34.2%)	85 (36.3%)	99 (41.8%)	101 (43.5%)	0.126
Drinking *n* (%)	72 (31.2%)	83 (35.5%)	90 (38%)	76 (32.8%)	0.422
DM family history *n* (%)	96 (41.6%)	79 (33.8%)	84 (35.4%)	83 (35.8%)	0.325
Thyroid nodule *n* (%)	190 (82.3%)	181 (77.4%)	183 (77.2%)	176 (75.9%)	0.362
HUA *n* (%)	60 (26%)	57 (24.4%)	45 (19%)	56 (24. 1%)	0.305
Hypertension *n* (%)	144 (62.3%)	134 (57.3%)	129 (54.4%)	126 (54.3%)	0.260
DR *n* (%)	64 (27.7%)	62 (26.5%)	66 (27.8%)	42 (18. 1%)^#^	0.044
DKD *n* (%)	80 (34.6%)	70 (29.9%)	62 (26.2%)	54 (23.3%)^#^	0.041
DSPN *n* (%)	84 (36.4%)	72 (30.8%)	62 (26.2%)	67 (28.9%)	0.105
CAS *n* (%)	159 (68.8%)	152 (65%)	144 (60.8%)	150 (64.7%)	0.341
LEAD *n* (%)	82 (35.5%)	89 (38%)	73 (30.8%)	83 (35.8%)	0.412
Insulin *n* (%)	58 (25. 1%)	57 (24.4%)	56 (23.6%)	48 (20.7%)	0.694
Metformin *n* (%)	50 (21.6%)	55 (23.5%)	45 (19%)	63 (27.2%)	0.195

FT3, free triiodothyronine; FT4, free thyroxine; FT3/FT4 FT3 to FT4 ratio; TSH, thyroid-stimulating hormone; Cr, creatinine; SUA, serum uric acid; eGFR, estimated Glomerular Filtration Rate; FPG, Fasting plasma levels ofglucose; TC, total cholesterol; TG, triglyceride; HDL, high-density lipoprotein; LDL, low-density lipoprotein; ALT, alanine aminotransferase; AST, aspartate aminotransferase; GGT, glutamyl transpeptidase; HbA1c, glycated hemoglobin A1c; UUAE,24 h urinary excretion of uric acid; SBP, systolic blood pressure; DBP, diastolic blood pressure; BMI, body mass index; DM, diabetes mellitus; HUA, hyperuricemia; DR, diabetic retinopathy; DKD, diabetic kidney disease; DSPN, diabetic sensorimotor polyneuropathy; CAS, carotid artery stenosis; LEA, lower extremity atherosclerosis.

Compared with Q1group, *p < 0.05; compared with Q2 group, ^#^*p* < 0.05; compared with Q3 group, ^&^*p* < 0.05.

### Relationships between FT3/FT4 ratio and other factors

Table 3 presents the correlations between the FT3/FT4 ratio and other variables. The FT3/FT4 ratio was weakly positively correlated with eGFR (*ρ* = 0.129, *P* < 0.001), TG (*ρ* = 0.081, *P* = 0.013), ALT (*ρ* = 0.103, *P* = 0.002), GGT (*ρ* = 0.121, *P* < 0.001), UUAE (*ρ* = 0.140, *P* < 0.001), DBP (*ρ* = 0.072, *P* = 0.028), and BMI (*ρ* = 0.108, *P* = 0.001). Weak inverse correlations were observed with HbA1c (*ρ* = −0.192, *P* < 0.001) and age (*ρ* = −0.115, *P* < 0.001). Although statistically significant, these correlations were small in magnitude.

**Table 3:** Relationships between FT3/FT4 ratio and other factors.

Factors	*r*	*p*
Age (years)	−0.115	*p* < 0.001
TSH (μIU/mL)	0.058	0.076
Cr (μmol/L)	−0.075	0.022
SUA (umol/L)	0.009	0.778
eGFR (mL/min/1.73m2)	0.129	*p* < 0.001
FBG (mmol/L)	−0.079	0.016
TC (mmol/L)	−0.061	0.06
TG (mmol/L)	0.081	0.013
HDL (mmol/L)	−0.041	0.216
LDL (mmol/L)	−0.029	0.371
ALT (U/L)	0.103	0.002
AST (U/L)	0.024	0.47
GGT (U/L)	0. 121	*p* < 0.001
HbA1c (%)	−0.192	*p* < 0.001
UUAE (mg/24 h)	0.140	*p* < 0.001
SBP (mmHg)	−0.009	0.782
DBP (mmHg)	0.072	0.028
BMI (kg/m2)	0.108	0.001

Figure 2 presents the FT3/FT4 ratio stratified by sex, age, BMI, and HbA1c. Women had a lower FT3/FT4 ratio than men (*P* < 0.01), whereas participants with HbA1c < 7% had a higher ratio than those with HbA1c ≥ 7% (*P* < 0.001). The FT3/FT4 ratio did not differ significantly between participants aged <45 and ≥45 years or between those with BMI <28 and ≥28 kg/m^2^ (both *P* > 0.05).

**Figure 2:**
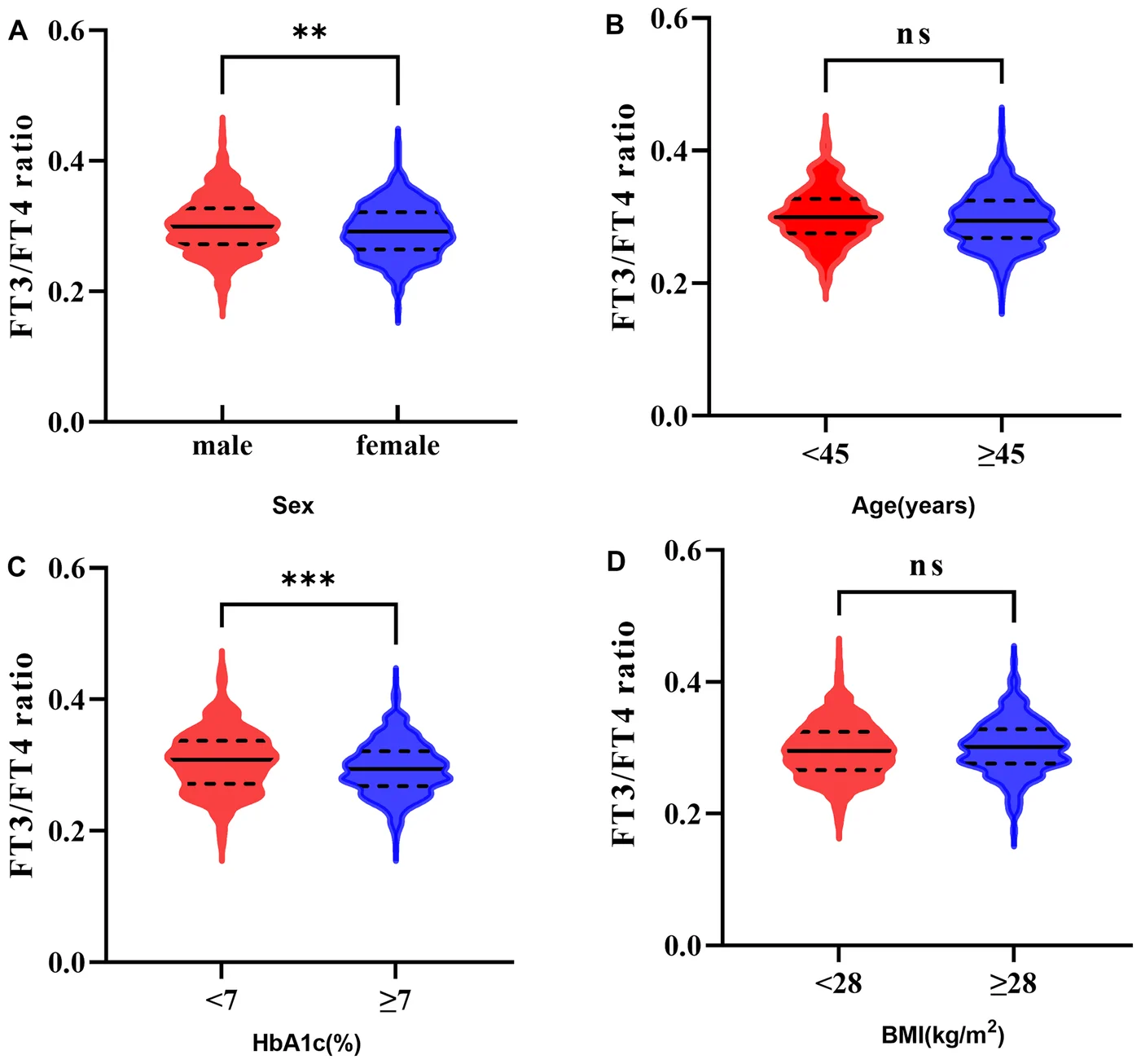
Distribution of the FT3/FT4 ratio according to sex, age, HbA1c, and BMI. **(A)** FT3/FT4 ratio according to sex (male vs. female). **(B)** FT3/FT4 ratio according to age (<45 vs. ≥45 years). **(C)** FT3/FT4 ratio according to HbA1c (<7% vs. ≥7%). **(D)** FT3/FT4 ratio according to BMI (<28 vs. ≥28 kg/m^2^). The solid lines indicate the median, and the dashed lines indicate the interquartile range. ns, not significant; **P < 0.01; ***P < 0.001.

### Association between FT3/FT4 ratio and thyroid nodule

In the unadjusted logistic regression model, each 0.1-unit increase in the FT3/FT4 ratio was associated with lower odds of TNs (OR = 0.65, 95% CI: 0.45–0.95; *P* = 0.027). This association was attenuated and no longer statistically significant after adjustment for age and sex (OR = 0.78, 95% CI: 0.52–1.17; *P* = 0.233). It remained nonsignificant after further adjustment for BMI, diabetes duration, smoking status, HbA1c, insulin use, and metformin use and TSH (OR = 0.77, 95% CI: 0.49–1.20; *P* = 0.249) (Table 4). No substantial multicollinearity was detected in the fully adjusted model, with tolerance values ranging from 0.657 to 0.948 and variance inflation factors ranging from 1.055 to 1.522. The Hosmer–Lemeshow goodness-of-fit test showed no evidence of poor model fit (*χ*^2^ = 6.144, df = 8, *P* = 0.631).

**Table 4:** Association between the FT3/FT4 ratio and the presence of thyroid nodules.

Model	Adjustment	OR (95% CI) per 0.1-unit increase in FT3/FT4 ratio	*P* value
Model 1	Unadjusted	0.65 (95% CI 0.45–0.95)	0.027
Model 2	Age and sex	0.78 (95% CI 0.52–1.17)	0.233
Model 3	Age, sex, BMI, diabetes duration, smoking status, HbA1c, insulin use, and metformin use, TSH	0.77 (95% CI 0.49–1.20)	0.249

ORs are presented per 0.1-unit increase in the FT3/FT4 ratio. In the fully adjusted model, tolerance values ranged from 0.657 to 0.948, and variance inflation factors (VIFs) ranged from 1.055 to 1.522, indicating no substantial multicollinearity. The Hosmer–Lemeshow goodness-of-fit test showed no evidence of poor model fit (χ^2^ = 6.144, df = 8, P = 0.631).

### Nonlinear association between the FT3/FT4 ratio and thyroid nodules

Restricted cubic spline analysis was used to assess the potential nonlinear association between the FT3/FT4 ratio and TNs. After adjustment for BMI, diabetes duration, smoking status, HbA1c, insulin use, metformin use, and TSH, the analysis showed a significant overall association (*P* for overall association = 0.036) and evidence of nonlinearity (*P* for nonlinearity = 0.022). Using the median FT3/FT4 ratio of 0.296 as the reference, the odds of TNs declined sharply as the ratio increased across its lower range. Above the reference value, the estimated odds increased modestly before returning toward the null at higher values. However, the 95% confidence intervals included 1.0 across most intermediate and higher values, indicating uncertainty in these estimates (Figure 3).

**Figure 3:**
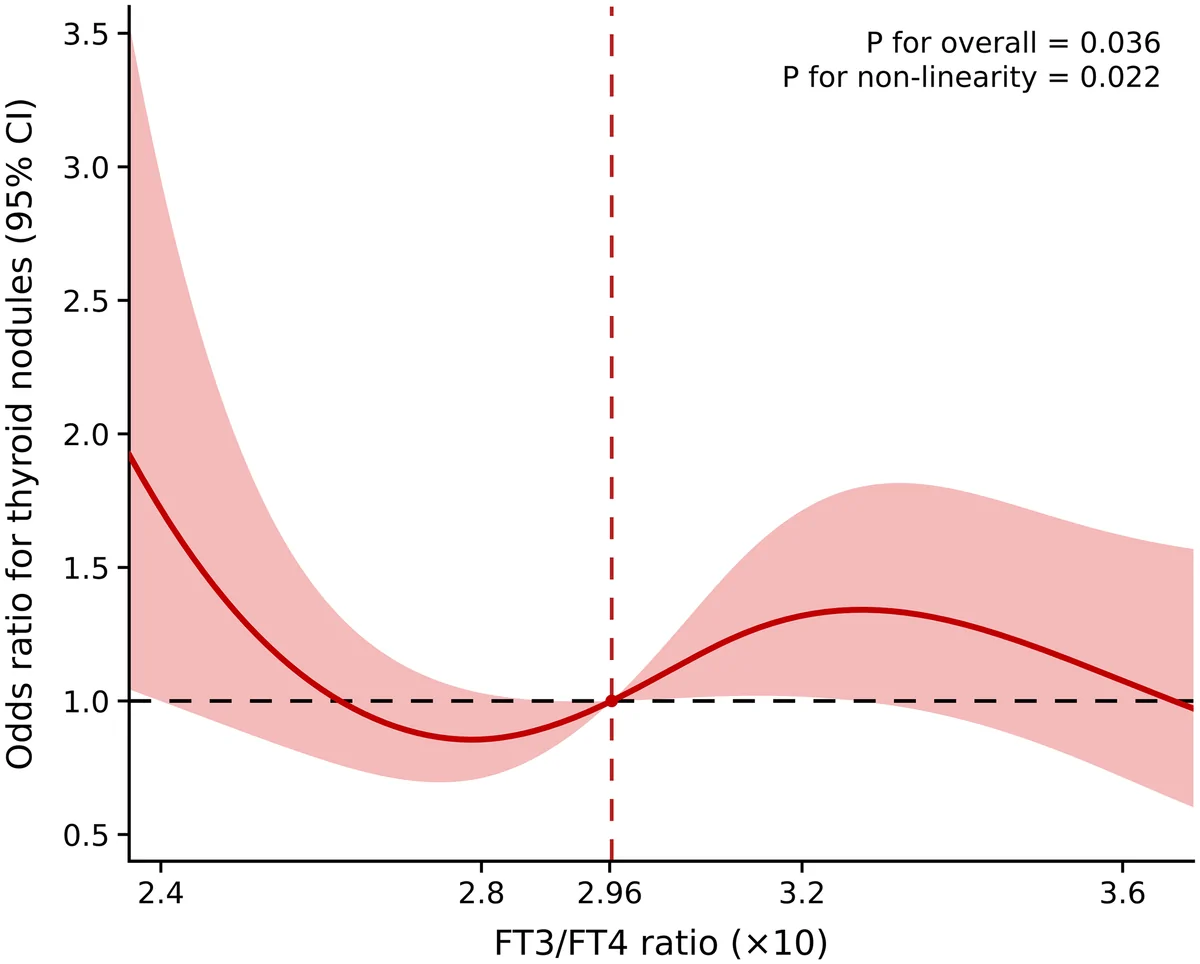
Restricted cubic spline analysis of the association between the FT3/FT4 ratio and the presence of thyroid nodules. The model was adjusted for age, sex, BMI, diabetes duration, smoking status, HbA1c, insulin use, and metformin use. The median FT3/FT4 ratio (0.296) was used as the reference. *P* for overall association = 0.036 and *P* for non-linearity = 0.022.

## Discussion

In this hospital-based cohort of euthyroid patients with T2DM, TNs were more prevalent in women than in men (84.2% vs. 73.9%), consistent with the findings of Cao et al. (21). The prevalence of TNs tended to decrease across increasing quartiles of the FT3/FT4 ratio, although the differences between quartiles were not statistically significant. In the unadjusted analysis, a lower FT3/FT4 ratio was associated with the presence of TNs. However, this association was attenuated after adjustment for age and sex and remained nonsignificant in the fully adjusted model that included TSH. These findings indicate that the crude association may be partly attributable to demographic and clinical factors.

Although conventional multivariable logistic regression did not identify a statistically significant linear association, restricted cubic spline analysis suggested a nonlinear association between the FT3/FT4 ratio and TNs (*P* for overall association = 0.036; *P* for nonlinearity = 0.022). Higher odds of TNs appeared to be concentrated at relatively low FT3/FT4 ratios, with little evidence of an association across the intermediate and higher ranges. A single linear effect estimate may therefore not adequately capture the shape of this association. However, the wider confidence intervals at the lower end of the distribution indicate greater uncertainty in this range. Given the cross-sectional design, this nonlinear pattern should be considered hypothesis-generating and requires confirmation in prospective studies.

The FT3/FT4 ratio is commonly used as an indirect marker of peripheral T4-to-T3 conversion, although its direction of change may vary according to the underlying physiological context. Higher FT3/FT4 ratios have been reported in individuals with obesity, fatty liver disease, and metabolic syndrome (22–24). In states of energy excess and insulin resistance, enhanced peripheral conversion of T4 to T3 may represent an adaptive response that promotes energy expenditure, consistent with the reported positive association between FT3 and BMI in euthyroid individuals (25, 26). By contrast, chronic inflammation and systemic illness may suppress deiodinase expression or activity, thereby reducing peripheral T3 production and lowering the FT3/FT4 ratio (27, 28). These mechanisms are not mutually exclusive, as patients with T2DM are metabolically heterogeneous and may simultaneously exhibit obesity-related adaptive conversion, insulin resistance, chronic inflammation, and comorbid illness. The observed FT3/FT4 ratio may therefore reflect the balance among these competing influences rather than a uniform effect of metabolic dysfunction (29).

This context-dependent interpretation may help explain previous reports of lower FT3/FT4 ratios in euthyroid men with TNs and in patients with papillary thyroid carcinoma (12, 13) with studies reporting higher ratios in obesity and metabolic syndrome. Inflammation has been implicated in thyroid tissue injury and nodule formation (21, 30, 31), and Inflammation-related suppression of peripheral T4-to-T3 conversion may therefore contribute to a lower FT3/FT4 ratio in some individuals with thyroid nodular disease. However, the presence of TNs does not itself indicate impaired deiodinase activity, and the reported associations may reflect differences in age, sex, metabolic status, comorbidity burden, or study population. In the present study, the inverse association between the FT3/FT4 ratio and TNs was observed only in the unadjusted analysis and was not significant in the fully adjusted linear model. The proposed metabolic and inflammatory mechanisms therefore remain speculative and cannot be established from our data, particularly because inflammatory biomarkers and deiodinase activity were not directly measured.

Previous studies have reported positive and negative associations of FT3 and FT4, respectively, with insulin resistance in euthyroid individuals with obesity (32). Obesity is also associated with alterations in thyroid function (33, 34), and FT3 has been positively correlated with BMI in euthyroid individuals with obesity and in patients with T1DM (35, 36). In our cohort, however, the FT3/FT4 ratio did not differ significantly between participants with BMI <28 and ≥28 kg/m^2^. This discrepancy may reflect differences in study populations, metabolic characteristics, definitions of obesity, or the relative contributions of obesity and inflammation to peripheral thyroid hormone metabolism. The cross-sectional design does not allow us to determine whether obesity or insulin resistance mediates the relationship between the FT3/FT4 ratio and TNs.

TNs were detected in 730 of 934 participants (78.2%), substantially exceeding the prevalence of 36.9% previously reported in the general Chinese population. However, these estimates are not directly comparable because our participants were hospitalized patients with T2DM rather than a community-based sample. They may have been older and had a greater burden of metabolic abnormalities and comorbidities associated with thyroid nodular disease. Thyroid ultrasonography may also have increased the detection of small or asymptomatic nodules. Differences in participant selection, age and sex distributions, ultrasound protocols, and diagnostic criteria may have further contributed to this discrepancy. Therefore, the observed 78.2% represents the proportion of participants with TNs in this selected hospital-based cohort and should not be interpreted as the population prevalence among all individuals with T2DM.

Several limitations should be acknowledged. First, the retrospective cross-sectional design precludes assessment of temporality or causality, and reverse causation cannot be excluded. Second, although glucose-lowering medication use was recorded, information on treatment duration, dosage, adherence, and other concomitant therapies was incomplete. Third, the single-center, hospital-based sample limits the generalizability of the findings to community-based populations, individuals without diabetes, and populations from other regions. Finally, inflammatory biomarkers and deiodinase activity were not measured, preventing direct evaluation of the proposed biological mechanisms.

## Conclusion

In euthyroid patients with T2DM, the FT3/FT4 ratio was not independently associated with TNs under a conventional linear assumption after multivariable adjustment. However, restricted cubic spline analysis suggested a possible nonlinear relationship, with higher estimated odds of TNs at lower FT3/FT4 ratios. Prospective studies are needed to confirm this finding and further clarify the relationship between peripheral thyroid hormone metabolism and TNs.

## Data Availability

The raw data supporting the conclusions of this article will be made available by the authors, without undue reservation.
